# Machine learning detection of heteroresistance in *Escherichia coli*

**DOI:** 10.1016/j.ebiom.2025.105618

**Published:** 2025-02-21

**Authors:** Andrei Guliaev, Karin Hjort, Michele Rossi, Sofia Jonsson, Hervé Nicoloff, Lionel Guy, Dan I. Andersson

**Affiliations:** a Department of Medical Biochemistry and Microbiology, Uppsala University, Uppsala, Sweden; b Department of Biosciences, University of Milan, Milan, Italy; c Department of Electronics, Information and Bioengineering, Politecnico di Milano, Milan, Italy; d SciLifeLab, Uppsala University, Uppsala, Sweden

**Keywords:** Antibiotic resistance, Antibiotic heteroresistance, *E. coli*, Machine learning, Piperacillin-tazobactam

## Abstract

**Background:**

Heteroresistance (HR) is a significant type of antibiotic resistance observed for several bacterial species and antibiotic classes where a susceptible main population contains small subpopulations of resistant cells. Mathematical models, animal experiments and clinical studies associate HR with treatment failure. Currently used susceptibility tests do not detect heteroresistance reliably, which can result in misclassification of heteroresistant isolates as susceptible which might lead to treatment failure. Here we examined if whole genome sequence (WGS) data and machine learning (ML) can be used to detect bacterial HR.

**Methods:**

We classified 467 *Escherichia coli* clinical isolates as HR or non-HR to the often used β-lactam/inhibitor combination piperacillin-tazobactam using pre-screening and Population Analysis Profiling tests. We sequenced the isolates, assembled the whole genomes and created a set of predictors based on current knowledge of HR mechanisms. Then we trained several machine learning models on 80% of this data set aiming to detect HR isolates. We compared performance of the best ML models on the remaining 20% of the data set with a baseline model based solely on the presence of β-lactamase genes. Furthermore, we sequenced the resistant sub-populations in order to analyse the genetic mechanisms underlying HR.

**Findings:**

The best ML model achieved 100% sensitivity and 84.6% specificity, outperforming the baseline model. The strongest predictors of HR were the total number of β-lactamase genes, β-lactamase gene variants and presence of IS elements flanking them. Genetic analysis of HR strains confirmed that HR is caused by an increased copy number of resistance genes via gene amplification or plasmid copy number increase. This aligns with the ML model's findings, reinforcing the hypothesis that this mechanism underlies HR in Gram-negative bacteria.

**Interpretation:**

We demonstrate that a combination of WGS and ML can identify HR in bacteria with perfect sensitivity and high specificity. This improved detection would allow for better-informed treatment decisions and potentially reduce the occurrence of treatment failures associated with HR.

**Funding:**

Funding provided to DIA from the 10.13039/501100004359Swedish Research Council (2021-02091) and NIH (1U19AI158080-01).

**Research in context**

**Evidence before this study**

Heteroresistance (HR) is a concerning phenotype characterized by a minor subpopulation of resistant bacterial cells present within a susceptible main population. This resistant subpopulation can lead to treatment failures as it can proliferate when the susceptible bacteria are killed by the antibiotic. Presently used antibiotic susceptibility tests have a very high error rate for detection of HR, resulting in frequent misclassification of HR isolates as susceptible. This misclassification can result in inappropriate treatment choices, contributing to persistent infections and the spread of resistance.

**Added value of this study**

The present study shows that HR in bacteria can be detected with 100% sensitivity and 84.6% specificity using a combination of whole genome sequence data and machine learning.

**Implications of all the available evidence**

This proposed approach drastically improves our ability to correctly identify HR isolates and patients with higher risk of treatment failure. This will allow precision diagnostics of HR and improved management of infections caused by these common bacteria.

## Introduction

Antibiotic resistance poses a threat to public health where *Escherichia coli* is a significant bacterial pathogen.^1^ *E. coli* is the most common cause of urinary tract infections and bloodstream infections in high-income countries,^2^ and the β-lactam/β-lactamase inhibitor combination TZP (piperacillin-tazobactam) is one of the most commonly used treatment options for bloodstream infections in Sweden. Recently the interest has increased regarding heteroresistance (HR), a difficult to detect type of resistance observed for several bacterial species and antibiotic classes.^3^ HR refers to a phenotype where a predominantly susceptible main population contains small subpopulations of resistant cells.^4^, ^5^, ^6^ A recently proposed criterion defines a bacterial isolate as HR if it contains a subpopulation at a frequency ≥10^−7^ that can survive at an antibiotic concentration 8-fold or above the highest antibiotic concentration not affecting the main population.^4^ Even though the clinical impact of HR remains unclear,^3^^,^^6^ mathematical models and animal experiments associate HR subpopulations with treatment failure.^7^, ^8^, ^9^, ^10^, ^11^ In addition, clinical studies of Gram-positive bacteria indicate that HR is associated with negative treatment outcomes whereas less support exists for Gram-negatives.^12^, ^13^, ^14^

At least two genetic mechanisms can cause HR: resistance mutations that often cause stable HR, or increased gene dosage of antibiotic resistance genes with low activity/expression through tandem gene amplifications, plasmid copy number increase or transposition of resistance genes to high-copy number plasmids. The latter type of HR is typically genetically unstable (i.e., in absence of selection the copy number is reduced) and these dynamics make HR a complex phenomenon to manage in clinical settings.^3^^,^^15^, ^16^, ^17^ HR due to increased gene dosage is prevalent in Gram-negative species and has been described for several species and antibiotic classes.^3^^,^^4^^,^^15^^,^^16^^,^^18^^,^^19^ Tandem gene amplifications in bacteria arise from homologous recombination between repeated sequences in sister chromatids during replication. Further unequal crossover events within the duplicated region can either lead to additional amplification or the loss of the duplication. Under antibiotic selective pressure, the number of amplified units can increase, and if the selective pressure is removed the amplifications are lost due to their intrinsic instability and fitness cost.^3^^,^^17^^,^^20^, ^21^, ^22^

Commonly employed phenotypic methods for heteroresistance detection include gradient diffusion strips (for example Etest), disc diffusion test, and PAP (population analysis profiling) test. Among these, the PAP test is considered the gold standard but it is labour-intensive and costly,^4^^,^^6^ whereas gradient diffusion strips and disc diffusion analyses exhibit low specificity and sensitivity for HR diagnostics.^4^^,^^5^^,^^14^^,^^18^^,^^22^ Since WGS of clinical isolates has become increasingly common as a tool to determine if bacterial strains are resistant to antibiotics, a number of studies focused on application of ML to WGS data in order to predict regular antibiotic resistance.^23^, ^24^, ^25^, ^26^ We employed various ML algorithms to detect TZP HR on a dataset consisting of 467 complete genomes of *E. coli*. Predictors employed by the best models correspond to known mechanisms and genetic requirements for increased gene dosage, and their performance surpasses that of a simple association-based method, demonstrating the utility of ML in HR detection.

## Methods

### Bacterial strain collection

A laboratory collection of 474 clinical blood stream infection *E. coli* isolated from 474 patients. The collection contains all blood stream infection *E. coli* isolates from 2014 to 2015 collected mainly from three different hospitals (Uppsala University hospital, Enköping and Östhammar hospitals) and a few samples from outpatient care within the Uppsala Region, Sweden. For 255 out of the 474 isolates clinical data are available.^27^

### Antibiotics and media

For broth as well as agar plates with and without antibiotic supplementation, Mueller-Hinton (Difco) media was used. Piperacillin and tazobactam were purchased from Sigma–Aldrich. The piperacillin-tazobactam (TZP) combination was used in an 8:1 ratio, and stated concentrations refer to piperacillin.

### Laboratory determination of HR phenotype

**Fig. 1:**
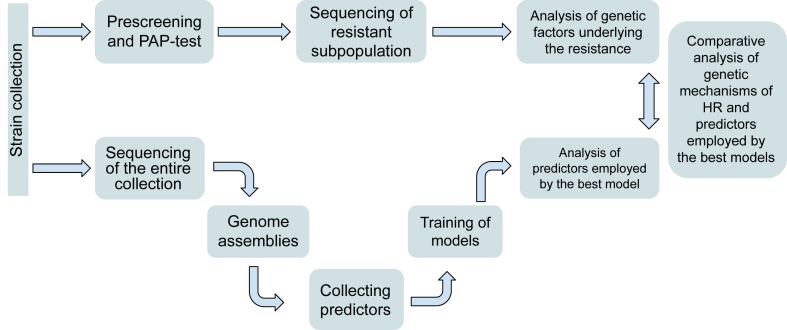
Overview of the analysis workflow.

A summary of the analysis workflow and methods is presented in Fig. 1. Isolates were screened for detection of TZP HR phenotype. To determine which isolates among the 474 clinical *E. coli* were HR, all strains were initially prescreened for growth on both 8 and 16 mg l^−1^ (clinical break point ≥16 mg l^−1^ TZP) TZP plates in duplicates. For the pre-screen an overnight culture was inoculated with one single colony into 1 ml of Mueller-Hinton (MH) media to be used as inoculum for a second over night culture, by adding 1 μl of the first overnight culture diluted a 1000-fold in MH media. From the second over night culture 100 μl was spread on agar plates (8 and 16 mg/L) with glass beads. Bacterial isolates without visible growth on 16 mg l^−1^ TZP were considered non-HR. For all *E. coli* isolates where a minimum of 1 colony could be detected on both 8 and 16 mg l^−1^ TZP plates for a minimum of 1 replicate (n = 123), a population analysis profile (PAP) test was performed in triplicates. For the PAP test an overnight culture was inoculated with one single colony in 1 ml of MH that was used as inoculum for a second over night culture by adding 1 μl of the first overnight culture diluted a 1000-fold. From the second over night culture 100 μl was spread on agar plates (8–128 mg/L) with glass beads. For concentrations below 8 mg/L overnight cultures were diluted in PBS before 5 μl drops were placed on Mueller-Hinton (MH) agar plates supplemented or not with TZP. Two-fold increments of antibiotics were used within the range of 0.25–128 mg l^−1^ TZP. Plates were incubated at 37 °C overnight (0.25–4 mg l^−1^ TZP) for 48 h (8–128 mg l^−1^ TZP). Population frequency was calculated using colony counts from plates without antibiotics as a baseline. Isolates displaying all the following criteria were considered HR: (i) a susceptible main population, (ii) growth of a resistant subpopulation on ≥16 mg l^−1^ TZP (EUCAST clinical breakpoint 2014 and 2015) with a ≥4-fold increase of resistance level compared to the unaffected main population (the less stringent criterion was used to include mutants with less increase in resistance) (iii) resistant subpopulation frequency of ≥10^−7^. These three criteria should be fulfilled for at least 2 out of 3 replicate experiments for an isolate to be included in the study as HR. Isolates not fulfilling these criteria were included as non-HR.

### Analysis of mutants

To determine the genetic mechanism behind the HR phenotype, resistant cells were selected on 4 and 8-fold the TZP MIC of the HR isolates (76 out of 80 isolates) on agar plates. The MIC for each HR isolate was determined with Etest (Etests from 2023, fixed tazobactam concentration of 4 mg/L). For the MIC determination an over night culture was diluted 1:20 (approx. 0.5 MacFarland) into 0.9% NaCl and spread with a steril cotton swab onto MH plates and an Etest was applied (bioMérieux). The MIC value of the main population was used as MIC value to calculate the TZP concentration for mutant selection. Four biological replicates of 100 μl overnight culture from each HR isolate were spread on MH agar plates supplemented with TZP at 4 and 8 × MIC with a 10 μl sterile loop. One mutant was selected from each plate, and whole genome sequencing was performed on one mutant per HR isolate predominantly selected on 8 × MIC (68/72 HR isolates selected from 8-fold), when no mutants (4/72 HR isolates) did grow on 8-fold MIC, mutants were isolated from 4-fold MIC. Each mutant was restreaked on an MH agar plate with the same TZP concentration and subsequently one colony was grown in 2 ml MH with the same TZP concentration. A cell pellet from 1 ml was saved at −20 °C for later DNA extraction with the MasterPure Complete DNA and RNA Purification kit (LGC Biosearch technologies) following the manufacturer's recommendations and 1 ml for storage at −80 °C.

### Sequencing and bioinformatic analysis

The parental strains were sequenced using both long-read Nanopore and short-read DNBseq technologies, while the resistant mutants were only sequenced with short-read DNBseq technology. For this, parental strains were isolated on MH agar plates and a single colony was used to start an overnight growth in 1.5 mL MH broth at 37 °C under vigorous shaking. DNA was extracted from 1 mL cultures using the MasterPure Complete DNA and RNA Purification kit (LGC Biosearch technologies) by following the manufacturer's recommendations with the following modifications: i) 0.5 μL of proteinase K was used instead of 1 μL, ii) 1.5 μL of RNAse A was used instead of 1 μL, iii) incubation with RNAse A was performed for 90' instead of 30' and included mixing of the samples by inverting them 10 times every 15'. Extracted DNA was quantified using Qubit 2.0 (Invitrogen) and the dsDNA broad range assay kit (Invitrogen). For Nanopore sequencing, DNA samples were diluted to 5.56 ng μl^−1^ and dilutions were checked again using the dsDNA high sensitivity assay kit (Invitrogen). Nanopore sequencing was performed in-house on a MinION Mk1C sequencer using R9 cells and the rapid barcoding kit 96 in order to multiplex 40 genomes per cell, and fast base calling was used. DNBseq was performed by BGI (Warsaw, Poland).

The sequencing reads were controlled for quality using FastQC v0.11.9 and MultiQC v1.12. Short reads were trimmed using fastp v0.20.1 and for Nanopore reads all the reads shorter than 3000 bp were discarded using Filtlong v0.2.1.^28^^,^^29^ The filtered reads were assembled with Unicycler v0.4.8 if Nanopore coverage was below 20×,^30^ otherwise with a custom pipeline consisting of Flye v2.9.1,^31^ medaka v1.6.0 and Polypolish v0.5.0,^32^^,^^33^ BWA v0.7.17 and seqkit v2.2.0.^34^^,^^35^ This pipeline starts with the Flye assembler, which takes the long reads and builds a draft genome sequence. The draft genome sequence undergoes a consensus improvement step using Medaka to refine the assembly. The draft is further enhanced using Polypolish where short reads are used to correct any remaining errors in the assembly. After genome assembly, all short reads were mapped to it and unmapped reads were collected to be submitted to SPAdes assembler in plasmid-assembly mode, to detect potential extra plamsids.^36^

Assemblies were automatically annotated using Prokka v1.14.6 and Prodigal v2.6.3 to identify protein-coding genes and tRNAscan-SE v2.0.9 to identify tRNA genes.^37^, ^38^, ^39^ The predicted protein sequences were analysed with RGI v5.2.0 and CARD v3.1.3 to identify putative resistance genes and mutations.^40^ Up- and downstream each resistance gene, a 100-kb region was retrieved using bedtools v2.30.0.^41^ Each 200-kb region surrounding a resistance gene was used as input to GRF v1.0 to find direct repeats (min length 20 bp, 0% mismatch).^42^ These regions were also processed by ISEScan v1.7.2.3 to find and classify insertion sequences (IS).^43^ All putative IS longer then 2000 nt were excluded from the analysis. Due to the stringency of our analysis (0% mismatch), long near-identical repeated sequences were detected as several smaller repeated sequences. Analysis of HR mutants included: (i) mapping short DNBseq reads from mutants onto parental genomes using bowtie2 v2.5.2,^44^ variant calling and filtering using bcftools v1.10,^45^^,^^46^ variant annotation using bedtools v2.31.1 and custom R scripts^41^; (ii) identification of amplified segments in the mutants’ genomes based on coverage change in 1 kb windows (deviations of greater than 2 standard deviations were taken into account) using bowtie2 v2.5.2,^44^ samtools v1.13,^46^^,^^47^ (iii) plasmid copy number change was inferred from short read coverage relative to chromosome coverage (using bowtie2 v2.5.2 and custom R scripts),^44^ and only an increase of 2-fold or more was taken into account; in addition, to rectify for amplifications on plasmid when calculating plasmid copy number increase any amplification on the plasmid was removed and the average sequence coverage was recalculated for the remaining plasmid sequence; (iv) IS transposition events were detected using ISmapper v2.0.2.^48^ Additionally, the CLC genomic workbench (Qiagen) was used to validate this analysis. Both pipelines were implemented in a Snakemake v7.24.0 and are freely available in the project’s repository on GitHub (https://github.com/andrewgull/HeteroR) along with other code,^49^^,^^50^ configuration files and tool options used for this study.

To assess the genetic diversity of *E. coli* strains used in this study, we built a phylogenetic tree using the core genome alignment of our strain collection (n = 474) together with 31 strains representing the major *E. coli* phylogenetic groups. The core-genome alignment was obtained using Roary v3.13.0.^51^ The tree was built with IQ-Tree v2.1.2 using maximum-likelihood analysis of a concatenated alignment of 2261 genes and the GTR + I + G model.^52^ The code for phylogenetic analysis is available in the project’s repository.

### Machine learning

#### Establishing set of predictors

From the genome assemblies, we collected predictors (or features) known or expected to be related to the described mechanisms of unstable HR,^3^^,^^53^ including presence of resistance genes (according to CARD database), presence of repeats around them, size of amplified region and technical artefacts that theoretically could affect the previous two categories.

First, the annotated resistance genes were filtered using the following criteria: (i) only strict and perfect hits were retained, (ii) only hits >90% identical to the reference sequence were retained, (iii) only hits covering at least 90% of the reference sequence were retained, (iv) resistance mechanisms like efflux, target alteration, permeability and SNPs were filtered out and (v) resistance mechanisms like antibiotic target replacement, antibiotic target protection, and antibiotic inactivation were retained.

Second, for each strain we constructed features related to resistance genes and repeat content. The main features were: (i) number and type of resistance genes, (ii) repeat count in a 200 kbp region surrounding a β-lactamase gene, (iii) total repeat length, (iv) average and maximum repeat length, (vi) total count of repeats longer than 100 bp, (vii) average length of amplifiable region (defined as the distance between direct repeats), (viii) total count of repeats longer than 500 bp, (ix) plasmid copy number inferred from the relative genome coverage and (x) presence of IS elements.

Third, some of the predictors were further split into more specific groups as: (i) presence of repeat on plasmids, on the chromosome or on the opposite sides of a resistance gene, (ii) number and length of repeats around *ampC*, *bla*_TEM_, *bla*_CTX_, and *bla*_SHV_ β-lactamase (BL) genes, (iii) total number of IS elements belonging to different families, (iv) presence of IS elements inside a resistance gene and (v) the average length of IS elements.

Fourth, we included features unrelated to mechanisms of gene amplifications that might affect the classification prediction: (i) chromosome assembly status (complete, i.e., circular, or not), (ii) Nanopore reads coverage and (iii) length of the longest Nanopore read.

#### Models and performance metrics

All modelling was done using *workflowsets* and *tidymodels* R packages.^54^, ^55^, ^56^ The ML algorithms we used were as follows: LASSO-regularized logistic regression (LLR)^57^^,^^58^; support vector machine with different kernel functions: linear, polynomial and radial basis (lSVM, pSVM and rbfSVM respectively)^59^; multilayer perceptron (MLP) and three ensemble methods: random forest (RF), gradient boosting trees (GBT) and bootstrap aggregated MLP.^60^, ^61^, ^62^

Our baseline model was derived from the fact that HR strains tend to encode more than 4 copies of β-lactamase genes, therefore the classification rule was formulated as follows: if a strain bears more than 4 β-lactamases, then it is classified as HR, non-HR otherwise.

We used area under the receiver operating characteristic (ROC) curve (AUC ROC) and Youden’s J-statistic as performance metrics to select the best hyperparameter configurations.^63^ Both metrics are robust to imbalanced data sets. ROC AUC can be seen as a probability that a randomly selected HR strain gets higher probability of being HR than a randomly selected non-HR strain and changes from 0.5 (classifier is as good as random guessing) to 1 (perfect classifier); J-statistic changes from 0 (classifier has no value) to 1 when both specificity and sensitivity reach their maximum. To choose the best preprocessing recipe within each ML algorithm family, we compared ROC-curve shape visually and mean ROC AUC values using Bayesian ANOVA using *tidyposterior* package which enabled us to make informed decisions based on the outcomes of the resampling procedure without the need to utilise the test data set.^64^

For final comparison of the best ML model with the baseline model on the test data set we used a number of metrics reflecting different sides of classifiers’ preformance. Besides AUC ROC, we used elemenatry (sensitivity, specificity, positive predictive value, negative predictive value) and composite performance metrics (Matthews correlation coefficient and Youden’s J-statistic), and performance measures based on probabilistic interpretation of error (mean absolute error [MAE], Cohen’s kappa) (for details see Supp. Appendix, statistical analysis).

To establish the importance of predictors employed by the best GBT model we used a permutation-based test for predictors’ importance with 1-AUC as the loss-function implemented in the R package DALExtra.^65^ During this test each predictor is withdrawn from the model, then the model’s performance is evaluated using AUC ROC and importance score *l* is calculated as *l* = *1 − AUC* resulting in more important predictors having higher importance score, because their withdrawal causes higher loss in performance compared to the full model. The whole process is repeated n times (in our case n = 500) for each predictor included in the model and median score is used for the final importance evaluation. For the LLR model, predictors importance was calculated as HR odds ratio via exponentiating each coefficient’s reciprocal.

#### Feature engineering

Feature engineering is a process of creating predictors or features from raw data and their transformation and scaling in order to improve a model’s ability to capture patterns in the data set. During this process, we obtained a table with a total of 122 predictors, referred to here as features (see ‘Methods: establishing set of predictors’). However, correlated predictors and excessive numbers of them can potentially create problems for some models. To overcome this, we employed several feature selection strategies.

First, for every algorithm, we eliminated predictors with near-zero variance to ensure a robust modelling process. Second, we introduced a correlation threshold as an additional hyperparameter to some of our models, ensuring the selection of uncorrelated predictors. Additionally, we utilised principal components generated through principal component analysis (PCA) instead of the original predictors.

For logistic regression, we employed the least absolute shrinkage and selection operator (LASSO) for feature selection. For other machine learning (ML) algorithms, we leveraged the Boruta algorithm of feature selection.^66^

To enhance the performance of non-decision tree-based models and PCA, we applied the Yeo-Johnson (YJ) transformation and ordered quantile normalizing transformation (ORQ) to the numeric variables.^67^^,^^68^ This transformation aimed to improve the normality and symmetry of the variable distributions.

Furthermore, to address the issue of imbalanced target variables, we incorporated the synthetic minority oversampling technique (SMOTE) during model training.^69^ This technique allowed us to generate synthetic samples and balance the representation of the target variable. As a result, we had 622 observations in the training data set evenly divided between HR and non-HR classes instead of the original 64:311 ratio.

Consequently, we trained each ML algorithm using the following preprocessing steps (referred to as recipes, according to tidymodels terminology): (i) *Basic recipe*: removing features with a near-zero variance; normalization of features; synthetic oversampling; (ii) *YJ recipe*: removing features with a near-zero variance; normalization of features; YJ-transformation of features; synthetic oversampling; (iii) *ORQ recipe*: removing features with a near-zero variance; normalization of features; ORQ-transformation of features; synthetic oversampling; (iv) Decorrelation filter recipe: recipes i-iii with a tunable decorrelation filter; (v) *PCA recipe*: removing features with a near-zero variance; feature normalization; features transformation using YJ-transformation; synthetic oversampling; feature compression using PCA; (vi) Base + Boruta recipe: removing features with a near-zero variance; normalization of features; synthetic oversampling; feature selection using the Boruta algorithm (for RF and BT algorithms).

#### Hyperparameters tuning and model validation

To fine-tune the hyperparameters for the GBT model, we employed space-filled grid search followed by Bayesian optimization using R-package *tune*; for the remaining models, we utilized a space-filled grid search using the same package.^70^ To evaluate the performance of the hyperparameter combinations, we conducted a 10-fold cross-validation repeated 10 times on 80% of the data set, performance evaluation was based on AUC ROC value. The remaining 20% of the data were never used during hyperparameter tuning, model training or selection. It was used only once for testing the best model's performance on unseen data. Splitting of the data set was done in a stratified way addressing the target variable imbalance.

### Statistical analysis

All statistical analysis was performed using R programming language,^54^ all plots were made using packages ggplot2,^71^ ggforce,^72^ ggpubr,^73^ ggbreak,^74^ and scales.^75^ Comparison of medians were performed using Kruskal–Wallis test, effects of phenotype HR/non-HR on count variables were measured using Poisson regression, effects of variables on phenotype – using Logistic regression. Results of the analysis are available in the Supplementary Appendix.

### Role of funders

The funding bodies had no say in data analysis, collection, interpretation, or decision to publish.

## Results

### Prevalence of heteroresistance

Our main hypothesis behind detection of HR using ML is that increased gene dosage is the dominant cause of HR for *β*-lactam antibiotics.^3^^,^^15^ To establish a set of HR and non-HR *E. coli* isolates for statistical analysis and modelling, 474 clinical *E. coli* bloodstream infections isolates were examined by pre-screening and PAP tests (see Materials and Methods) to determine if they were HR or non-HR against TZP (Fig. 1). After exclusion of 5 resistant strains and 2 potentially misidentified strains (see next section), prevalence of TZP HR was 17% (80/467) among the isolates.

### Phylogenetic analysis

To validate the genetic diversity of our entire collection, we conducted a phylogenetic analysis of the core genome alignment (number of genes = 2328) of the 474 genomes of clinical *E. coli* bloodstream infection isolates together with 31 strains representing major clades of *E. coli* (Supplementary Fig. S1 and Table S1).^76^ The resulting phylogram demonstrates that our collection covers these clades, but with an overrepresentation of isolates from the B2 clade. HR strains were distributed over the entire tree, representing all the clades, showing that HR was not associated with any specific *E. coli* clade. Two strains DA63246 and DA63068 clustered with the outgroup species *E. albertii* and *E. fergusonii* and were therefore excluded from the further analysis along with 5 resistant strains. Thus, we proceeded with a dataset of 467 strains that included 387 non-HR and 80 HR complete genomes for feature engineering, model training, validation and testing.

### β-lactamase genes and plasmids

**Fig. 2:**
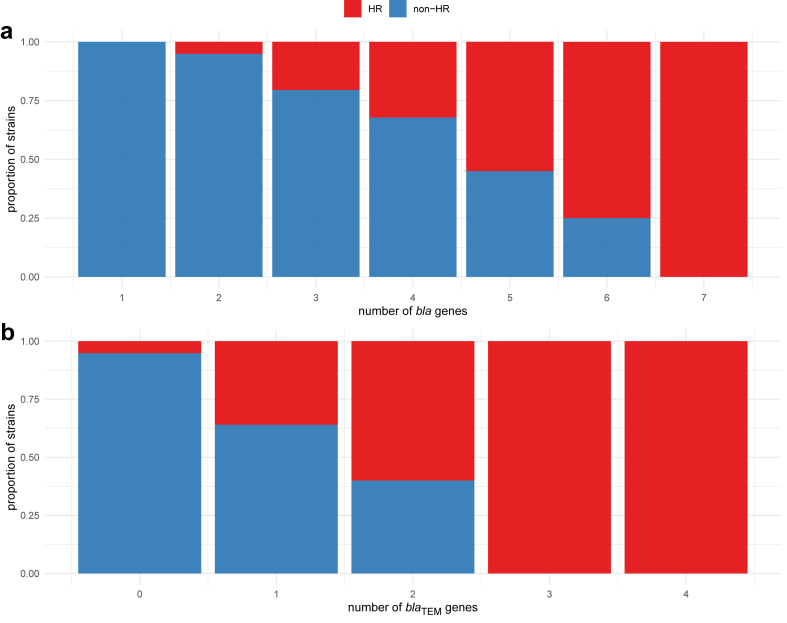
Association between the number of *bla* genes and HR phenotype. (**a**) Proportions of HR and non-HR phenotypes in strains with different numbers of *bla* genes (*bla*_CTX-M,_*bla*_TEM,_*bla*_OXA,_*bla*_SHV_). (**b**) Proportions of HR and non-HR phenotypes in strains with different numbers of *bla*_TEM_ genes.

Since we hypothesized that amplification of β-lactamase genes is one of the mechanism causing HR to TZP, we counted β-lactamase genes (different β-lactamase genes together with copies of the same β-lactamase gene) in all the assembled genomes. The median number of β-lactamase genes per strain was 3 (minimum 1 and maximum 7) and the majority of isolates harbouring more than 4 β-lactamase genes were HR (Fig. 2a). This observation formed our baseline predictive model: if a strain has more than 4 β-lactamase genes, it is likely to be an HR strain. Furthermore, regression analysis showed that the total number of β-lactamase genes served as a significant predictor of HR, with each additional β-lactamase gene reducing the likelihood of non-HR phenotype by a coefficient of 0.37 (Logistic regression, p ≪ 0.001). Additionally, only HR strains were found to carry 3 *bla*_TEM_ genes, and the majority of strains carrying 2 *bla*_TEM_ genes were also HR (Fig. 2b). Moreover, HR strains demonstrated a significantly higher number of plasmids compared to non-HR strains (Poisson regression, p ≪ 0.001). Thus, the number of β-lactamase genes is strongly associated with the HR phenotype and an increased number of these genes increases the likelihood of a strain becoming HR.

### Perfect tandem repeats

**Fig. 3:**
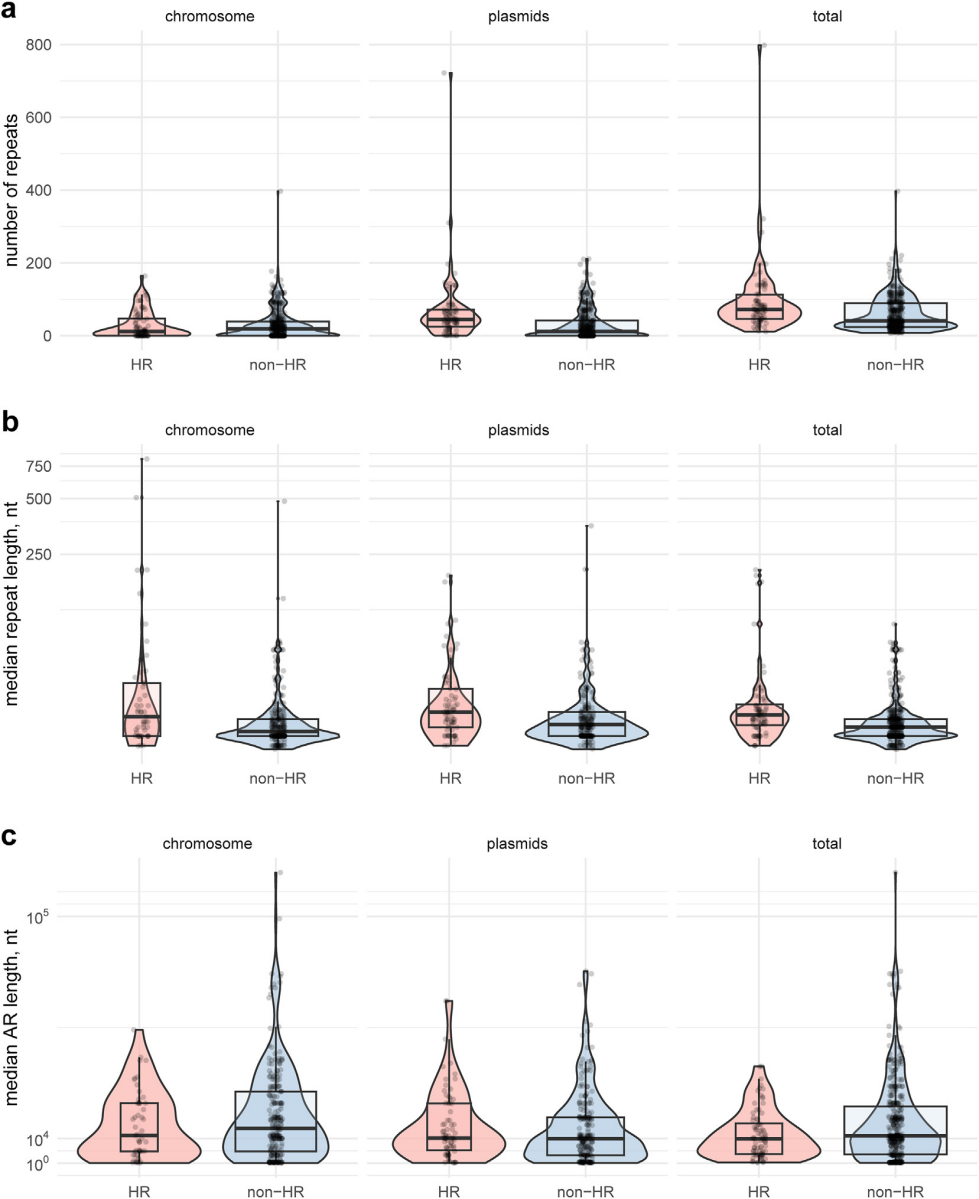
Number and location of repeats in HR (n = 80) and non-HR strains (n = 387). (**a–c**) Box- and violin plots of the number of direct repeats, median repeat length and median amplifiable region length in HR and non-HR strains depending on their location (plasmids, chromosomes or both). The box represents the interquartile range (IQR), with the centre line indicating the median. The IQR captures the middle 50% of the data, with 25% of the data points falling below the box and 25% above it. The whiskers extend from the box to show the range of the remaining data within the 1.5 IQR value. Overlayed violin plots show the shape of distribution of the data. nt: nucleotides.

Tandem repeats flanking the resistance gene(s) are typically required for homologous recombination to generate the amplifications and the resulting increase in β-lactamase gene copy number.^21^^,^^53^^,^^77^ The number of direct repeats (≥20 nucleotides, 0% mismatch) detected within 100 kb of any β-lactamase gene was significantly different between HR and non-HR isolates where non-HR isolates showed a decrease in repeat count by a coefficient of 0.63 (p-value ≪ 0.001, Poisson regression, Supplementary Appendix). The median number of direct repeats was 72 in HR strains compared to 42 in non-HR strains. Moreover, only HR strains exhibited isolates with more than 400 repeats (Fig. 3a). Also, HR strains displayed a greater median repeat length compared to non-HR strains (34 bp vs. 29 bp, Kruskall–Wallis test, p-value < 0.001, Supplementary Appendix, statistical analysis) (Fig. 3b). Overall, these findings indicate that HR strains exhibit distinct patterns in terms of repeat content, including longer repeat lengths, a higher occurrence of long repeats, and an increased abundance of direct repeats near a β-lactamase gene.

### Amplifiable region length

We defined the amplifiable region (AR) as a region containing a β-lactamase gene, flanked by two direct DNA repeats. We hypothesized that a shorter AR facilitates amplification, as the fitness costs are expected to increase with increased AR size and associated alterations in levels of RNAs or proteins that could lead to imbalances in gene dosage^17^^,^^22^^,^^78^ However, our results did not support this hypothesis: for AR on plasmids and chromosomes separate or altogether, the median AR length did not differ significantly between HR and non-HR strains, although the longest AR are present mainly in non-HR strains (Fig. 3c).

### Insertion sequence content

**Fig. 4:**
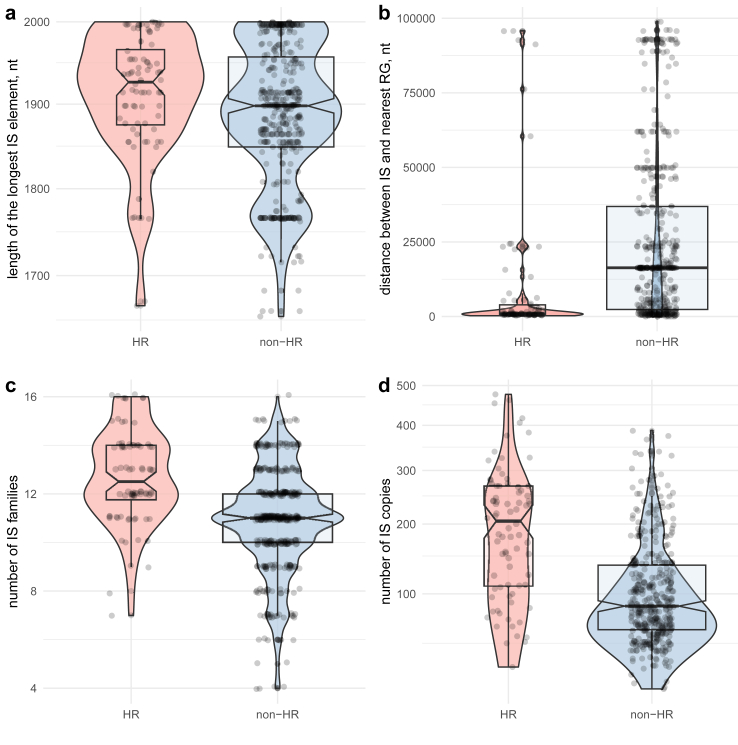
Length, location, family and copy number of the IS-elements in HR (n = 80) and non-HR (n = 387) strains. (**a–d**) Box- and violin plots of the longest IS-element, the distance between IS and the closest *bla* gene, the number of different IS families found within each strain, the total number of IS-elements found in each strain respectively. The box represents the interquartile range (IQR), with the centre line indicating the median. The IQR captures the middle 50% of the data, with 25% of the data points falling below the box and 25% above it. The whiskers extend from the box to show the range of the remaining data within the 1.5 IQR value. Overlayed violin plots show the shape of distributions of the data. RG: resistance gene, nt: nucleiotides.

Insertion sequences are often present at several copies in a genome and are an important source of long repeated sequences. Such repeats have previously been shown to provide substrate for unequal crossing-over events that generate amplification of resistance genes and thereby cause heteroresistance.^3^^,^^53^ We hypothesized that their prevalence is associated with HR phenotype. In line with this assumption, we observed the following: (i) certain IS families exhibit a notable prevalence in HR strains, namely IS1380, IS6, and IS1182 (Supplementary Fig. S2), (ii) the maximum length of IS sequences is slightly longer in HR strains compared to non-HR (1926 nt compared to 1898 nt, Kruskall–Wallis test, p = 0.01, Supplementary Appendix, statistical analysis) (Fig. 4a), (iii) the median distance between insertion sequences and the closest *β*-lactamase gene is significantly shorter in HR strains than in non-HR (905 nt vs. 16,339 nt, Kruskall–Wallis test, p-value ≪ 0.001, Supplementary Appendix, statistical analysis) (Fig. 4b), (iv) the median count of IS families in HR strains is higher than in non-HR (12.5 and 11.0 families), and each additional IS family decrease the chance of exhibiting non-HR phenotype by a coefficient of 0.86, Poisson regression, p ≪ 0.001, Supplementary Appendix) (Fig. 4c), (v) the median number of IS copies per strain is higher in HR strains compared to non-HR (205 vs. 86, Kruskall–Wallis test, p-value ≪ 0.001, Supplementary Appendix, statistical analysis) (Fig. 4d). The high number of IS in both groups is likely to be inflated due to the fact that IS elements were counted in 100-kb regions around each *bla* gene without correction for overlaps between the regions. This explanation is supported by the correlation between number of *bla* genes and number of IS copies per strain (Spearman’s ρ = 0.57, p-value ≪ 0.001, Supplementary Appendix, statistical analysis). These results show that HR strains have more variants and a higher abundance of IS elements and more IS families.

### Machine learning based prediction of HR

We compared the performance of 7 supervised ML algorithms (see Methods), followed by comparison of the best one to the ‘4 β-lactamases’ (4BL) baseline model. Every ML algorithm showed moderate to very good performance on the training data set with mean ROC AUCs ranging from 0.843 to 0.900. Notably, models with principal components as predictors performed well (mean ROC AUC 0.843–0.876) with only 1 to 3 PCs used as predictors (Supplementary Table 2). Based on the results of Bayesian ANOVA, we chose the GBT model with Bayesian optimisation of hyperparameters (significantly better than any other model) and the LASSO Logistic Regression (LLR) model with YJ-transformation of predictors (the second-best model, but simpler, more generalizable and easier to interpret) (Supplementary Fig. 3a and b and Table S3). We fitted both the LLR and GBT models on the entire training data set and then applied them to the test data set (20% of the original data) which represented the unseen data. On the test data set the ROC AUC values of both models increased (0.905 for LLR, and 0.922 for GBT) indicating very good performance and no overfitting in both cases. These two best models were compared to the baseline 4BL model. Both GBT and LLR demonstrated a far better performance than the 4BL model in every metric except specificity (Table 1).

**Table 1:** Comparison of performance metrics of the baseline model (train and test data sets) and the two best machine learning algorithms (only test data set).

Metric^a^	Baseline model (train/test)	LLR	GBT
ROC AUC	–	0.877	0.935
Sensitivity, true positive rate (TPR)	0.266/0.063	0.813	1.000
Specificity	0.971/0.974	0.808	0.846
Positive predictive value (PPV)	0.654/0.333	0.464	0.571
Negative predictive value (NPV)	0.865/0.835	0.955	1.000
Matthews correlation coefficient (MCC)	0.350/0.079	0.510	0.695
Youden’s J statistic	0.236/0.037	0.620	0.846
Cohen’s κ	0.309/0.054	0.478	0.652
Mean absolute error (MAE)	0.150/0.181	0.201	0.135

a Regarding metrics see Methods section ‘Models and performance metrics’.

### Genetic mechanisms of HR

Previous work showed that the main mechanisms causing HR towards β-lactam antibiotics are tandem amplifications of β-lactamase genes, increased plasmid copy number of plasmids with β-lactamase genes and/or transposition events of β-lactamase genes to high copy number plasmids where the increased gene dosage leads to resistance.^3^^,^^15^^,^^17^^,^^18^ To determine the underlying genetic mechanisms of the HR phenotype for these TZP HR strains, resistant mutants were isolated from 72 out of 80 HR subpopulations (8 isolates were not examined because resistant mutants could not be obtained or sequence data was of poor quality), selected at 4 or 8-fold above MIC. Three major mechanisms, all leading to increased gene dosage were detected (Supplementary Fig. S4 and Table S4). Tandem gene amplification of *β*-lactamase genes (*bla*_TEM_, *bla*_CTX-M_, *bla*_OXA_) was the predominant mechanism displayed by 45.8% (33/72) of the HR *E. coli* isolates. The repeat sequence at the end points of the amplification was dominated by IS26 (IS6 family) insertion sequences involved in 66.7% (24/36) of the tandem gene amplifications. The size of the amplified unit varied from 2 kb to 32 kb and the copy number of amplified units varied between 2 and 54. The second-most common genetic mechanism was increased copy number of plasmids carrying *β*-lactamase genes (*bla*_TEM_, *bla*_CTX-M_, *bla*_OXA_) (20.8%, 15/72), where the plasmid copy number increase varied between 2- to 31-fold. The insertion of a plasmid containing a *β*-lactamase gene (*bla*_TEM_ or *bla*_OXA_) into a 16 or 23S rRNA gene with the insertion point on the plasmid located in front of the *β*-lactamase gene on the plasmid was the third most common mechanism (8.3%, 6/72). In 7% (5/72) transposition of a *bla*_TEM_ containing transposon to one or multiple plasmids with higher copy number was observed. The remaining isolates displayed a mix of tandem amplification/plasmid copy number increase (4.2%, 3/72), mutations (4.2%, 3/72) or one cases where the mechanism remains unknown. In conclusion, the dominating genetic mechanism behind the HR phenotype involves increased dosage of *β*-lactamase genes via tandem amplifications, plasmid copy number increase and transposition events, either individually or in combination.

## Discussion

Heteroresistance complicates antibiotic treatment due to the difficulties in detecting the rare and often unstable resistant subpopulations using existing antimicrobial susceptibility testing methods.^4^ Here, we show that machine learning methods can successfully detect unstable HR to TZP in *E. coli* based on genomic sequencing data. Out of 7 ML algorithms examined, we chose the two best-performing models, GBT and LLR, and compared their performance with a simple deterministic model based on an empirical rule: if a strain contains more than 4 *β*-lactamase genes it is classified as HR and otherwise non-HR.

While GBT achieved good general performance as reflected by the area under the ROC curve (ROC AUC 0.935, Table 1) without overfitting, LLR offered a simpler alternative with slightly lower performance (ROC AUC 0.877). The other metrics values (such as sensitivity, specificity etc., Table 1) depend on the classification threshold, i.e., the probability value produced by a model which we consider high enough (see below) to assign a given sample to the HR class (Supplementary Fig. S5). We used the maximum value of Youden’s J statistic as the point corresponding to the classification threshold maximizing the sensitivity and specificity for our unbalanced data set. For the LLR model the classification threshold was at 0.29 and for the GBT model it was set at 0.02 (Supplementary Fig. S5).

The baseline model had, by design, high specificity (0.971; 97.1% of strains classified as HR were actually HR), but this came at the cost of low sensitivity (0.266; the method only classified 22.5% of all HR strains as HR). GBT and LLR balanced these metrics better: with good specificity (0.846 and 0.808 for GBT and LLR, respectively), they achieved a near-perfect or perfect sensitivity (0.813 for LLR and 1.0 for GBT model, respectively). Cohen's κ statistic, which considers the number of correct classifications adjusted for unequal size of HR and non-HR classes, also reflected the superiority of the ML models. The baseline model achieved a mere κ = 0.309 on the train data set, while LLR reached κ = 0.478 (moderate) and GBT reached κ = 0.652 (substantial) on the test data set.^79^ On the other hand, the baseline model showed a higher precision or PPV (0.654), indicating fewer false positives, compared to the ML models which achieved a moderate precision (0.464 and 0.571, LLR and GBT correspondingly). Youden’s J statistic (0.620 and 0.846 for LLR and GBT, respectively) confirmed the advantage of these models over the baseline (J = 0.236) on the unbalanced dataset.^80^ Similarly, the Matthews correlation coefficient indicated a moderate correlation between the models' predictions and actual classifications (0.510 and 0.695 for LLR and GBT, respectively) compared to the baseline (0.350).^81^ Finally, the MAE – a metric independent of the chosen classification threshold that shows how much the predicted HR probabilities differ from the actual probability (P_HR_ = 1) – demonstrated that the best model was GBT (MAE = 0.135), followed by the baseline model (MAE = 0.150), and by the LLR with MAE = 0.201.

In conclusion, GBT offered the best overall accuracy, sensitivity, negative predictive value and J-index, outperforming LLR in every perfomance measure and outperforming the baseline model in critical metrics. Nevertheless, the LLR model was still better than the baseline model in important metrics, offering a good balance between complexity and flexibility.

**Fig. 5:**
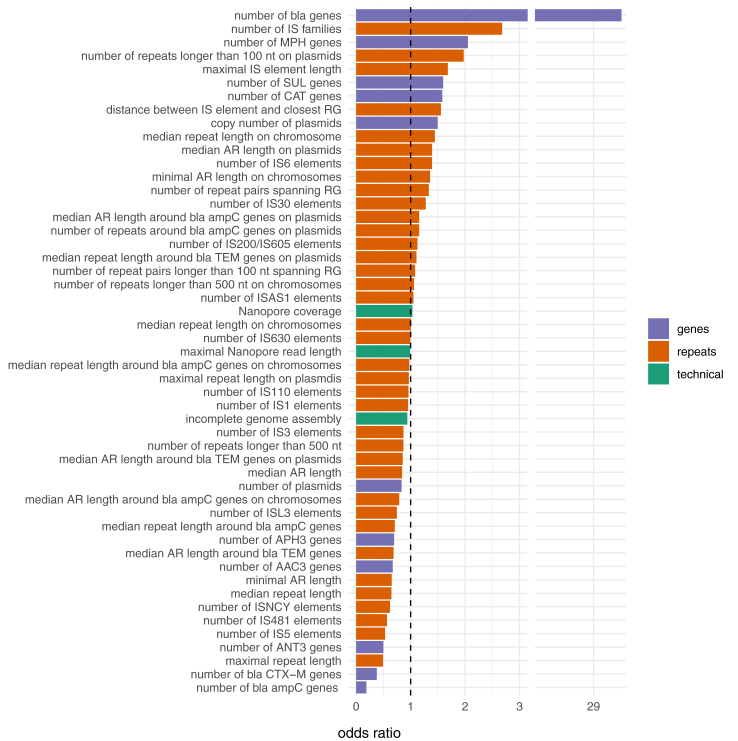
Predictors employed by the best model and their effects: odds ratio of the features present in the LLR model. Odds ratio (OR) is the ratio of the probability of a strain being classified as HR to the probability of the same strain being classified as non-HR. OR below 1 (dashed vertical line) indicate predictors decreasing the probability of a strain being classified as HR, and OR greater than 1 indicate predictors increasing the probability of a strain being classified as HR. The bars are coloured according to the group of features they represent: related to gene content (purple), to repeat content (orange) and technical artefacts (green). RG: resistance genes, AR: amplifiable region.

To assess the importance of the predictors employed by the LLR model, we exponentiated each coefficient’s reciprocal (51 predictors, Fig. 5). Features increasing the probability of HR can be divided into three groups: resistance genes, IS elements and other repeats, and plasmids. The total number of β-lactamase genes was the most important factor affecting the decision taken by the model, which is expected because β-lactamases are the main source for TZP resistance. The same applies to predictors related to repeats and insertion sequences. Repeats are a source of homology for unequal sister chromatid recombination which is the main mechanism of amplifications of genome segments. Alternatively, transposons can transfer β-lactamase genes to other locations of the genome, and the most common IS element in our collection of strains, IS26 from the IS6 family, is known to transpose in *E. coli*, transferring resistance genes to different locations on the genome.^82^ Specifically, it has been shown that IS26 transferred *bla*_TEM-1B_ and *bla*_TEM-1_ genes and that the transposition led to resistance and HR to TZP in *E. coli,*.^83^ However, IS26 was not part of the transposition events observed within our mutant collection or in a previous study by Nicoloff et al., 2024, instead Tn2 or Tn3 was observed.^15^ Furthermore, these and other IS elements can create new promoters upon insertion that could upregulate neighbouring resistance genes.^84^ The IS30 family is also known to cause DNA rearrangements in bacteria due to its transpositional activity.^85^ IS30 was not overrepresented in our HR isolates but was chosen by the model as an important predictor, probably because in certain combinations with IS6 it improves the quality of the prediction. Chloramphenicol acetyltransferase genes (*cmlA6*, *catI* and *cat3B*) are predictors likely due to their co-localization with *bla*_CTX-M_, *bla*_OXA_ and *bla*_TEM_.

Predictors decreasing HR probability fell into three categories. The first group includes repeats (median repeat length) and IS elements (IS3, IS1, IS110, IS5, ISL3, IS481 and ISNCY) and AR length. In the analysis of HR mechanisms, we found that the repeats leading to tandem amplifications were dominated by IS26 (IS6 family) elements (66.7%) whereas IS3, IS110, IS481 or ISNCY were not involved. The second group comprised resistance genes such as *bla*_CTX-M_ and *bla*_ampC_, ANT(3) (integron-encoded aminoglycoside acetyltransferase), APH(3) (aminoglycoside O-phosphotransferase), AAC(3) (aminoglycoside N-acetyltransferase). Within our set of HR mutants there were never any amplifications of *ampC* genes and the absence of repeats near the *ampC* gene on the chromosome can explain why they were in the non-HR category. The reason for why *bla*_CTX-M_ is a predictor for non-HR strains is more difficult to explain. However, strains with only *bla*_CTX-M_ and no other β-lactamase genes (*bla*_TEM_, *bla*_OXA_, *bla*_SHV_) were often non-HR (7/9 strains) in our mechanistic study and only one HR strain showed an amplification of *bla*_CTX-M_ alone as an mechanism behind HR (Supplementary Fig. S4). Finally, sequencing quality (Fig. 5) can affect HR prediction due to potential assembly errors. Thus, incomplete assemblies can lead to overestimation of plasmids and underestimation of repeats, thereby introducing noise in the data set.

In contrast to the LLR model, for the GBT model the importance of the predictors is represented by distributions, which makes it harder to interpret their exact role in the final classification. The most important predictor for the GBT model (Supplementary Fig. S6) was the median repeat length around *bla*_TEM_ genes located on plasmids with median loss *l* = 0.007, followed by maximum IS element length, maximum repeat length on plasmids and number of *bla* genes (loss *l* = 0.006, 0.004 and 0.004 respectively). Less important predictors were number of *bla*_TEM_ genes, median AR length on plasmids and number of IS6 elements (loss *l* = −0.014, −0.005, −0.004, respectively). Overall, this analysis shows that different architectures of models lead to emphasis on different predictors.

The prevalence of TZP HR in our study (17%) was higher than observed in a previous study of 423 clinical isolates of *Klebsiella pneumonia* (6%)^18^ and for other β-lactam/β-lactamase combinations in a study of HR prevalence for newer β-lactam/β-lactamase inhibitors (2–13%) in carbapenem resistant Enterobacterales.^86^ It can be noted that the prevalence frequencies determined by PAP tests could be affected by our use of a fixed ratio between piperacillin:tazobactam as compared to using a fixed concentration of tazobactam. The prevalence presented here is determined by PAP analysis with agar plates containing a fixed 8:1 ratio of piperacillin:tazobactam to mimic the concentrations used during treatment. However, this might in theory have lead to an increased HR prevalence in comparison to using a fixed concentration of tazobactam. We analysed this possibility by extracting MIC values from our PAP curves (where ratio is fixed) and compared this to the E-test MIC values (fixed concentration of tazobactam). This comparison shows that above 3 mg/L of piperacillin there is no difference in MIC values (within one concentration step, Supplementary Fig. S7). Since our PAP test defines a strain as being HR at or above the clinical breakpoint of 16 mg/L the tazobactam concentrations used in our PAP tests are either at 2 or 4 mg/L, which is near or at the recommended tazobactam concentration of 4 mg/L. Thus, it is unlikely that our use of a fixed ratio has any substantial effects on the screen positive rate.

It should also be noted that the different cutoff values used for defining a strain as HR might vary between different researchers and regulatory committees (EUCAST vs. CLSI), and that this can have an impact on whether strains are classified as HR or non-HR. This is a concern, especially in clinical settings where misclassification could be problematic. In this study we used the following HR definition: frequency of resistant subpopulation ≥10^−7^, increase in resistance level as compared to the susceptible population ≥4-fold and clinical breakpoint of TZP resistance ≥16 mg/L. However, for example, if a lower clinical breakpoint (>8 mg/L), was used this would lead to an increased TZP HR prevalence but also that more HR isolates would be classified as resistant rather than susceptible. Similarly, changing the cutoff of the resistant subpopulation from 10^−7^ to 10^−6^ would result in a reduced HR prevalence. At present, one cannot say that one cutoff value is more correct than the other since they are not yet linked to clinical outcome parameters. An important future objective is to determine which cutoff values are the most relevant for assessing the risk of worse clinical outcome.^27^

In accordance with earlier studies, the dominant mechanisms behind TZP HR identified here were increased gene dosage of β-lactamase genes by either tandem amplifications of β-lactamase genes, copy number increases of plasmids carrying β-lactamase genes, transposition of β-lactamase genes to other plasmids.^15^^,^^18^ In addition, we also detected plasmids with β-lactamase genes inserted into a 23 or 16S rRNA gene, which leads to TZP resistance through increased gene dosage of β-lactamase genes, similarly to tandem gene amplifications of *bla*_TEM_.^87^

This study applies ML in order to detect unstable TZP HR in *E. coli*. However, ML might also be applied for other antibiotics and bacterial species where unstable HR are the mechanism behind HR. For Gram-negative bacteria unstable HR is the most common mechanism behind HR and previous studies have detected unstable HR in *E. coli*, *Salmonella* Typhimurium, *Klebsiella pneumoniae*, *Acinetobacter baumannii* and also in the Gram-positive bacterium *Enterococcus faecium* for ertapenem, ceftazidime/avibactam, piperacillin/tazobactam, cefiderocol and cefepime (beta-lactamase genes), tigecycline (*tet*A), amikacin and netilmicin (*aac(6’)-Ib-cr*), trimethoprim/sulfamethoxazole (*sul1*), colistin (*arnD*) and vancomycin (*vanM*) due to tandem amplifications, plasmid copy number increases and/or transposition event of resistance genes to high copy number plasmids.^3^^,^^4^^,^^15^^,^^16^^,^^18^^,^^19^^,^^88^^,^^89^ ML has also recently been applied to genomic data in order to identify HR in *Candida parapsilosis* to micafungin.^90^ For *C. parapsilosis* ten genomic features were identified which can be rapidly measured by Sanger sequencing or quantitative PCR, allowing ML models to detect HR strains of *C. parapsilosis* as precisely as phylogeny-based prediction (sensitivity 0.70). Our study is different from Zhai et al.^90^ in that we have a larger data set and higher performance of the models (both higher sensitivity and specificity). Nevertheless, both studies show the utility of ML to detect HR from genomic data in different types of organisms and antimicrobial compound.

A demonstration of the relevance of detecting TZP HR in clinical settings was recently published for parts of this strain collection (255 out of the 467 strains) that was included in a retrospective study on patient treatment out come. In this retrospective study, an increased relative risk (odds ratio 3.1) for admittance to an intermediate care unit associated with pip-taz HR when the patient was treated with TZP. Similarly, an increased risk of transfer to an ICU (odds ratio 5.6) and mortality (odds ratio 7.1) was associated with gentamicin HR.^27^

We acknowledge some limitations of our study. First, the dataset size was relatively small which can impact the generalizability of the findings. Second, we studied bloodstream *E. coli* isolates collected from a limited geographical area and time period. Third, the dataset suffered from class imbalance: the HR target class contained only 80 strains, while the non-HR class contained 387 strains. Therefore, comprehensive capture of statistical features specific to HR strains could have been hindered. Despite attempts to mitigate this issue through oversampling techniques and alternative performance metrics, the problem remained partially unresolved.^69^ Fourth, the detection of repeated sequences was not perfect in our study. The method we used involved counting pairs of identical DNA segments or IS elements in overlapping genomic regions (100 kb up- and down-stream of resistance genes), resulting in a higher count of repeats compared to the actual number of individual repeats present in the genomes. These limitations may have influenced the accuracy, representativeness, and completeness of the results. Further research with larger and more balanced datasets, different drugs and bacterial species, refined testing approaches, and improved repeat detection methodologies is warranted to address these limitations.^3^

In summary, our study demonstrates that ML algorithms based on a limited set of sequence features can be used to detect unstable HR to TZP in clinical *E. coli* isolates with perfect sensitivity and good specificity. The identified predictors shed light on the potential genetic and sequencing-related factors that may promote or reduce the emergence of HR in bacteria via increased dosage of resistance genes. Understanding these positive and negative associations will aid the development of more accurate models for HR prediction in clinical settings, and improve HR detection to allow precision treatment of infected patients.

## Contributors

Conceptualisation: AG, LG, DIA.

Data curation: AG, KH, SJ, MR, HN.

Formal analysis: AG, KH, SJ, MR, HN.

Funding acquisition:DIA.

Investigation: AG, KH, SJ, MR, HN.

Methodology: AG, KH, MR, SJ, HN.

Project administration: DIA.

Supervision: DIA.

Verification of the underlying data: KH, HN, SJ.

Writing – original draft: AG, KH, DIA.

Review & editing: all authors.

All authors read and approved the final version of the manuscript.

## Data sharing statement

All sequenced strains were deposited in the NCBI database under the bioprojects PRJNA1165464 (474 parental isolates) and bioprojects PRJNA1083935 and PRJNA1160527 (HR mutants). The features table used for modelling is available at the project’s GitHub repository https://github.com/andrewgull/HeteroR/notebooks/modelling/data. All code necessary for reproducing the analysis, including software settings and is available at the GitHub repository https://github.com/andrewgull/HeteroR.

## Declaration of interests

The authors declare that they have no known competing financial interests or personal relationships that could have appeared to influence the work reported in this article.
