# Supplementary material for: Machine learning detection of heteroresistance in Escherichia coli
Source: eBioMedicine. 2025 Feb 21;113:105618. doi: 10.1016/j.ebiom.2025.105618 (PMC11893328; doi:10.1016/j.ebiom.2025.105618)
Supplement: Supplementary Appendix, Figs. S1–S7, and Tables S1–S3 [file mmc1.docx]

**Supplementary Appendix**

**Machine learning detection of heteroresistance in *Escherichia coli***

Andrei Guliaev, Karin Hjort, Michele Rossi, Sofia Jonsson, Hervé Nicoloff, Lionel Guy and Dan I. Andersson

Table of Contents

[Supplementary Figures 3](#_Toc181556362)

[Supplementary Tables 10](#_Toc181556363)

[Statistical analysis 12](#_Toc181556364)

[**Effect of number of beta-lactamases on resistance: Logistic regression** 12](#_Toc181556365)

[**Effect of resistance on number of plasmids: Poisson regression** 12](#_Toc181556366)

[**Effect of resistance on plasmid copy number: Poisson regression** 12](#_Toc181556367)

[**Effect of resistance on number of direct repeats: Poisson regression** 12](#_Toc181556368)

[**Median repeat length: Kruskal-Wallis rank sum test** 12](#_Toc181556369)

[**Effect of resistance on IS family count: Poisson regression** 13](#_Toc181556370)

## **Supplementary Figures**

Fig. S1 Phylogram of 474 *E. coli* strains used in this study and 31 strains from Dunne et al^76^ representing different *E. coli* clades. *E. albertii* and *E. fergusonii* were used as outgroup. The phylogram was built using a core genome alignment consisting of 2328 genes.

Fig. S2 Proportions of HR and non-HR phenotypes among strains carrying different families of IS-elements.

Fig. S3 Comparison of the best models from each group of ML algorithms used in this study. **a** ROC curves of the best models; **b** posterior distribution of ROC AUC of the best models. Model abbreviations: GBT: gradient-boosted trees, MLP BAG: bagged multilayer perceptron, LLR: LASSO logistic regression, poly SVM: polynomial support vector machine, linear SVM: linear support vector machine, rbf SVM: support vector machine with radial basis function.

Fig. S4 β-lactamase genes (*bla*_TEM_, *bla*_OXA_, *bla*_CTX-M_ and *bla*_SHV_) present in heteroresistant (HR), resistant and non-HR isolates. *Isolates that do not contain any of the *bla*_TEM_, *bla*_OXA_, *bla*_CTX-M_ and *bla*_SHV_ genes.

Fig. S5 Correction of the probability threshold according to the Youden’s J-index in both LR and GBT models. Vertical dashed line marks the classification threshold (0·29 for LLR, 0·02 for GBT) corresponding to the maximum J-statistic (0·620 for LLR, 0·846 for GBT).

Fig. S6 Importance of the predictors used by the GBT model.

Fig. S7 Comparison of MIC determined by agar MIC with fixed ratio of piperacillin:tazobactam (TZP) and MIC determined by Etest (fixed tazobactam concentration at 4 mg/L) for heteroresistant (HR) isolates (DA numbers are in house strain numbers).

## **Supplementary Tables**

| Isolate/Strain | NCBI RefSeq assembly |
| --- | --- |
| NCTC86EC | GCF_900092615.1 |
| ATCC 35469 (*E. fergusonii*) | GCF_000026225.1 |
| ABU 83972 | GCF_000148365.1 |
| APEC 01 | GCF_000014845.1 |
| CFT073 | GCF_014262945.1 |
| IAI39 | GCF_000026345.1 |
| O26:H11 str. 11368 | GCF_000091005.1 |
| S88 | GCF_000026285.1 |
| ED1a | GCF_000026305.1 |
| O157:H7 str. Sakai | GCF_000008865.2 |
| UMN026 | GCF_000026325.1 |
| O111:H str. 11128 | GCF_000010765.1 |
| REL606 | GCF_000017985.1 |
| SMS-3-5 | GCF_000019645.1 |
| E24377A | GCF_000017745.1 |
| SE15 | GCF_000010485.1 |
| SE11 | GCF_000010385.1 |
| KF1 (*E. albertii*) | GCF_000512125.1 |
| 55989 | GCF_000026245.1 |
| CB9615 | GCF_000025165.1 |
| E2348/69 | GCF_000026545.1 |
| IAI1 | GCF_000026265.1 |
| 042 | GCF_000027125.1 |
| HS | GCF_000017765.1 |
| 536 | GCF_000013305.1 |
| O103:H2 str 12009 | GCF_000010745.1 |
| ETEC H10407 | GCF_000210475.1 |
| W | GCF_000184185.1 |
| UTI89 | GCF_000013265.1 |
| K12 MG1655 | GCF_000005845.2 |

Table S1. Reference strains used for phylogenetic analysis and their RefSeq accession numbers.

| **Model type** | **Preprocessing recipe** | **mean ROC AUC** | **ROC AUC SE** | **mean J-index** | **J-index SE** |
| --- | --- | --- | --- | --- | --- |
| LLR | BASE | 0,8803441 | 0,006297784 | 0,5199831 | 0,01981194 |
| **LLR** | **BASE + YJ** | **0,886128** | **0,006525043** | **0,5878756** | **0,01912957** |
| LLR | BASE + ORQ | **0,89049** | 0,006555434 | 0,5780768 | 0,01983609 |
| linear SVM | NCORR | 0,8781521 | 0,006680676 | 0,5314316 | 0,01918198 |
| linear SVM | NCORR + YJ | 0,8706946 | 0,006553994 | 0,5530891 | 0,01906354 |
| linear SVM | NCORR + ORQ | 0,8800108 | 0,006737114 | 0,5597957 | 0,01969876 |
| linear SVM | PCA | 0,847437 | 0,008532648 | 0,5564977 | 0,01719619 |
| polynomial SVM | NCORR | 0,8855773 | 0,006132058 | 0,5367281 | 0,0204092 |
| polynomial SVM | NCORR + YJ | 0,8798525 | 0,005872618 | 0,6057235 | 0,01784356 |
| polynomial SVM | NCORR + ORQ | 0,8889798 | 0,006384383 | 0,5591106 | 0,01954465 |
| polynomial SVM | PCA | 0,8535466 | 0,007794472 | 0,5396144 | 0,01819229 |
| radial basis function SVM | NCORR | 0,8645151 | 0,007511995 | 0,5232965 | 0,01989775 |
| radial basis function SVM | NCORR + YJ | 0,8626718 | 0,07335926 | 0,5595499 | 0,01690254 |
| radial basis function SVM | NCORR + ORQ | 0,8662468 | 0,007316768 | 0,5655115 | 0,01860154 |
| radial basis function SVM | PCA | 0,8550835 | 0,008273015 | 0,5781321 | 0,01865395 |
| MLP | NCORR | 0,8888794 | 0,006928832 | 0,4892243 | 0,02057192 |
| MLP | NCORR + YJ | 0,87181 | 0,006278996 | 0,4944777 | 0,01964265 |
| MLP | NCORR + ORQ | 0,8750812 | 0,006152969 | 0,4953748 | 0,01844396 |
| MLP | PCA | 0,8571457 | 0,00724475 | 0,55098 | 0,0186876 |
| MLP + Bagging | NCORR | 0,8993781 | 0,005759025 | 0,5031167 | 0,01979609 |
| MLP + Bagging | NCORR + YJ | 0,8825358 | 0,006130091 | 0,5073825 | 0,01843576 |
| MLP + Bagging | NCORR + ORQ | 0,8865865 | 0,006422816 | 0,5244716 | 0,0209569 |
| MLP + Bagging | PCA | 0,8598047 | 0,007231239 | 0,5240323 | 0,02183251 |
| RF | BASE | 0,8932426 | 0,006882875 | 0,4986343 | 0,02299196 |
| RF | BASE + BORUTA | 0,890077 | 0,006104679 | 0,5136578 | 0,0196627 |
| GBT | BASE | 0,8892271 | 0,006338906 | 0,4807926 | 0,01971828 |
| **GBT + Bayesian opt.** | **BASE** | **0,9018561** | **0,00579141** | NA | NA |
| GBT | BASE + BORUTA | 0,8924053 | 0,006070261 | 0,4692949 | 0,01942803 |

Table S2. Performance comparison of different models and preprocessing recipes used in this study. The intensity of color reflects the cell's value: darker color means higher value.

| contrast | probability | mean | lower | upper | size |
| --- | --- | --- | --- | --- | --- |
| GBT vs MLP | 0.9974 | 0.015161678088465783 | 0.006529877143631166 | 0.023833587558145283 | 0 |
| LR vs GBT | 0.0016 | -0.01614410225049487 | -0.02463261823151412 | -0.0075289839901981365 | 0 |
| LR vs MLP | 0.4276 | -9.824241620290852e-4 | -0.009541577770443163 | 0.007524710937245695 | 0 |

Table S3. Results of Bayesian ANOVA for contrasting of the models resamples.

Table S4. Mechanisms of HR in the mutants – see file Supplementary table S4.xlsx

## **Statistical analysis**

### **Effect of number of beta-lactamases on resistance: Logistic regression**

| Coefficients | Estimate | exp(estimate) | Std. Error | z-value | Probability > \|z\| |
| --- | --- | --- | --- | --- | --- |
| Intercept | 4.6400 | 103.5462539 | 0.4537 | 10.226 | < 2e-16 |
| Number of β-lactamase genes | -1.0047 | 0.3661428 | 0.1331 | -7.549 | 4.39e-14 |

Null deviance: 427.73 on 466 degrees of freedom

Residual deviance: 357.41 on 465 degrees of freedom

AIC: 361.41

### **Effect of resistance on number of plasmids: Poisson regression**

| Coefficients | Estimate | Std. Error | z-value | Probability > \|z\| |
| --- | --- | --- | --- | --- |
| Intercept | 1.17865 | 0.06202 | 19.005 | < 2e-16 |
| Resistance type: non-HR | -0.33357 | 0.07040 | -4.738 | 2.15e-06 |

Null deviance: 927.39 on 466 degrees of freedom

Residual deviance: 906.32 on 465 degrees of freedom

AIC: 2005.2

### **Effect of resistance on plasmid copy number: Poisson regression**

| Coefficients | Estimate | exp(Estimate) | Std. Error | z-value | Probability > \|z\| |
| --- | --- | --- | --- | --- | --- |
| Intercept | 2.59432 | 13.3875000 | 0.03056 | 84.902 | < 2e-16 |
| Resistance type: non-HR | -0.33100 | 0.7182063 | 0.03468 | -9.545 | < 2e-16 |

Null deviance: 8479.7 on 466 degrees of freedom

Residual deviance: 8394.1 on 465 degrees of freedom

AIC: 9815.8

### **Effect of resistance on number of direct repeats: Poisson regression**

| Coefficients | Estimate | exp(Estimate) | Std. Error | z-value | Probability > \|z\| |
| --- | --- | --- | --- | --- | --- |
| Intercept | 4.54143 | 93.8250000 | 0.01154 | 393.46 | < 2e-16 |
| Resistance type: non-HR | -0.4651 | 0.6280316 | 0.01331 | -34.96 | < 2e-16 |

Null deviance: 18106 on 466 degrees of freedom

Residual deviance: 16981 on 465 degrees of freedom

AIC: 19651

### **Median repeat length: Kruskal-Wallis rank sum test**

| Chi-squared | 28.553 |
| --- | --- |
| Degrees of freedom | 1 |
| p-value | 9.117e-08 |

Median repeat length HR = 33.75

Median repeat length non-HR = 29.00

**Maximum IS length:** **Kruskal-Wallis rank sum test**

| Chi-squared | 6.6151 |
| --- | --- |
| Degrees of freedom | 1 |
| p-value | 0.01011 |

Max IS length HR = 1926.5

Max IS length non-HR = 1898.0

**Minimum distance from IS to the gene: Kruskal-Wallis rank sum test**

| Chi-squared | 51.869 |
| --- | --- |
| Degrees of freedom | 1 |
| p-value | 5.934e-13 |

Median distance HR = 905.5

Median distance non-HR = 16339.0

### **Effect of resistance on IS family count: Poisson regression**

| Coefficients | Estimate | exp(Estimate) | Std. Error | z-value | Probability > \|z\| |
| --- | --- | --- | --- | --- | --- |
| Intercept | 2.54160 | 12.7000 | 0.03137 | 81.013 | < 2e-16 |
| Resistance type: non-HR | -0.15125 | 0.8596 | 0.03494 | -4.329 | 1.5e-05 |

Null deviance: 211.72 on 466 degrees of freedom

Residual deviance: 193.56 on 465 degrees of freedom

AIC: 2182

**IS copy number per strain:** **Kruskal-Wallis rank sum test**

| Chi-squared | 60.047 |
| --- | --- |
| Degrees of freedom | 1 |
| p-value | 9.262e-15 |

IS copy number HR = 205

IS copy number non-HR = 86

**Correlation between number of *bla* genes and number of IS copies per strain: Spearman’s rank correlation**

S = 7242869, p-value < 2.2e-16

alternative hypothesis: true rho is not equal to 0

sample estimates: ρ = 0.5733092
